# Implicit Perception of Differences between NLP‐Produced and Human‐Produced Language in the Mentalizing Network

**DOI:** 10.1002/advs.202203990

**Published:** 2023-02-07

**Authors:** Zhengde Wei, Ying Chen, Qian Zhao, Pengyu Zhang, Longxi Zhou, Jiecheng Ren, Yi Piao, Bensheng Qiu, Xing Xie, Suiping Wang, Jia Liu, Daren Zhang, Roi Cohen Kadosh, Xiaochu Zhang

**Affiliations:** ^1^ Department of Psychology School of Humanities & Social Science University of Science & Technology of China Hefei Anhui 230026 China; ^2^ Department of Radiology the First Affiliated Hospital of USTC School of Life Science Division of Life Science and Medicine University of Science & Technology of China Hefei 230027 China; ^3^ Computational Bioscience Research Center (CBRC) King Abdullah University of Science and Technology (KAUST) Thuwal 4700 Saudi Arabia; ^4^ Centers for Biomedical Engineering School of Information Science and Technology University of Science & Technology of China Hefei Anhui 230027 China; ^5^ Microsoft Research Asia Beijing 100080 China; ^6^ Philosophy and Social Science Laboratory of Reading and Development in Children and Adolescents (South China Normal University) Ministry of Education Guangzhou 510631 China; ^7^ State Key Laboratory of Cognitive Neuroscience and Learning Beijing Normal University Beijing 100875 China; ^8^ Faculty of Health & Medical Sciences University of Surrey 30AD04 Elizabeth Fry Building Guildford GU2 7XH UK; ^9^ Application Technology Center of Physical Therapy to Brain Disorders Institute of Advanced Technology University of Science & Technology of China Hefei 230026 China

**Keywords:** human language, implicit perception, mentalizing network, natural language processing

## Abstract

Natural language processing (NLP) is central to the communication with machines and among ourselves, and NLP research field has long sought to produce human‐quality language. Identification of informative criteria for measuring NLP‐produced language quality will support development of ever‐better NLP tools. The authors hypothesize that mentalizing network neural activity may be used to distinguish NLP‐produced language from human‐produced language, even for cases where human judges cannot subjectively distinguish the language source. Using the social chatbots Google Meena in English and Microsoft XiaoIce in Chinese to generate NLP‐produced language, behavioral tests which reveal that variance of personality perceived from chatbot chats is larger than for human chats are conducted, suggesting that chatbot language usage patterns are not stable. Using an identity rating task with functional magnetic resonance imaging, neuroimaging analyses which reveal distinct patterns of brain activity in the mentalizing network including the DMPFC and rTPJ in response to chatbot versus human chats that cannot be distinguished subjectively are conducted. This study illustrates a promising empirical basis for measuring the quality of NLP‐produced language: adding a judge's implicit perception as an additional criterion.

## Graphical abstract

The study reveals that the subjectively undetectable differences between natural language processing (NLP)‐produced and human‐produced language are detected by the mentalizing network, which illustrates a promising empirical basis for measuring (and thus engineering) ever‐higher quality NLP‐produced language: adding a judge's neural activity as a criterion when assessing and optimizing NLP.

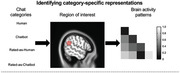

## 1 Introduction

Language is central to all aspects of social communication in humans, and the research field of natural language processing (NLP) encompasses computational techniques for learning, understanding and producing human language content.^[^ ^1^ ^]^ Therefore, NLP is the core in our communication with machines and also informs our understanding of communication among ourselves. Extensive ongoing efforts in NLP research have been made to produce human‐quality language. Although huge advances have been achieved, there are nontrivial challenges in methods to assess the quality of NLP‐produced texts.

Extant studies on NLP have focused on developing or improving the ability of programs to interpret and respond meaningfully to human language.^[^ ^2^ *^–^* ^4^ ^]^ However, it is highly conspicuous that very few published studies have considered the assessment criteria for language quality for the judge who assesses the quality of NLP‐produced texts. To our understanding, the current assessment is to use self‐reporting as the criterion of judgment.^[^ ^4^ *^–^* ^7^ ^]^ Traditionally, researchers have relied on self‐report measures to assess a person's demographic information, beliefs, and feelings.^[^ ^8^, ^9^ ^]^ However, self‐report measures are subjective and insensitive.^[^ ^10^ ^]^ For example, it is known that when assessing sensitive issues such as racial discrimination,^[^ ^11^ ^]^ contraception use^[^ ^12^ ^]^ and sexual orientation,^[^ ^13^ ^]^ self‐report measures are subject to both recall bias and social desirability bias. Furthermore, self‐report measures are insensitive and consequently not suited to assess appraisals of ambiguous situations, automatic attention bias, and reflexive psychopathological symptoms.^[^ ^14^ *^,^* ^15^ ^]^ Thus, the new assessment criteria for judging may involve the implicit measures that are commonly considered objective and relatively sensitive.^[^ ^16^ ^]^

Psycholinguistics research has demonstrated that the language we use in our daily lives can reveal aspects of our social and psychological worlds,^[^ ^17^ ^]^ supporting that language encodes information relevant to fields including personality, social, clinical and cognitive psychology, and it is thought that the social and psychological functions of language are processed by a reader or listener mainly at a low or implicit level.^[^ ^17^ *^,^* ^18^ ^]^ Thus, implicit perception might be highly informative for measuring the quality of NLP‐produced language.

Psycholinguistic evidence has shown that a person's language usage patterns are stable across time and context.^[^ ^17^ *^,^* ^19^ ^]^ Researchers analyzed a large body of text samples taken from journal abstracts, college writing assignments, and diaries across days and even years, and detected good internal consistency (across text types) for dozens of linguistic dimensions.^[^ ^20^ ^]^ Another study sampled students’ natural conversations and showed a relative stability of linguistic features across context: there were no significant linguistic difference effects when language was assessed in the diverse contexts, including home versus public places, workplaces versus coffee shops and direct personal interactions versus phone interactions.^[^ ^21^ ^]^ Although language use is stable within an individual, individuals differ in the ways they talk and write. Even when the content of the message is the same, individuals express themselves with their own distinctive styles. Language usage patterns have been recognized as stamps of individual identity.^[^ ^20^ ^]^ Of note, individual differences in language usage patterns have been demonstrated as related to individual differences in personality traits.^[^ ^20^ ^]^

In contrast, the language usage patterns of NLP systems might be unstable. Designing NLP systems with consistent personality—reflected by stable and consistent language usage patterns or language style—is a reported approach for establishing an emotional connection with users, although this remains a large challenge.^[^ ^22^ ^]^ NLP‐produced language is typically generated by retrieval‐based or generation‐based approaches,^[^ ^23^ ^]^ with both approaches modeling language over very large datasets based on language materials from different sources and people.^[^ ^23^ ^]^ An NLP system's verbal response to a user's utterance already exists in the training datasets, and is selected by algorithms. Thus, the NLP system's responses are derived from multiple people, suggesting the consequence that NLP‐produced language represents multiple personalities.^[^ ^24^ ^]^ In the last decade, models that can learn to control the style and usage patterns in language generation have been proposed,^[^ ^24^ *^–^* ^26^ ^]^ which has almost certainly improved the perceived quality of NLP‐produced language. However, the NLP systems have as‐yet not reached the ability to produce language with language usage patterns as stable as humans.^[^ ^23^ ^]^ Thus, we hypothesize that the variance of personality perceived from NLP‐produced language is larger than that from human‐produced language.

Neural activity data is more sensitive to the processes of psychological cognition than behavioral data.^[^ ^27^ *^,^* ^28^ ^]^ Therefore, we propose that adding neural activity data from a judge who assesses the quality of NLP‐produced texts as an implicit index to the assessment criteria for language quality could substantially improve our ability to reliably determine whether the NLP‐produced language achieves the human quality.

Social competence, defined as the ability to interact effectively with others while reaching personal goals, has been conceptualized as “effectiveness” in social interactions.^[^ ^29^ ^]^ People perceive the social competence of non‐human agents based on aspects such as personality, physical attributes, and emotional traits.^[^ ^30^ ^]^ Specific to the NLP systems, the social competence inferred from their produced language can contribute to establishing an emotional connection with users.^[^ ^25^ ^]^ During a conversation, people can purposefully and automatically infer others’ mental states, even with virtual agents’,^[^ ^31^ ^]^ which has been shown to engage the mentalizing network.^[^ ^32^ ^]^

Mentalizing, a fundamental component of human social cognition, describes the process by which we understand the assumed inner thoughts and intentions of others by observing or imagining others’ actions, speech, facial expressions, etc.^[^ ^33^ ^]^ The reported neural basis of mentalizing (i.e., the mentalizing network) includes the temporoparietal junction (TPJ) and the medial prefrontal cortex, among other regions.^[^ ^34^ ^]^ Understanding the communicative intent of an utterance requires the mentalizing network to be invoked to progress from coded meaning to speaker meaning.^[^ ^18^ ^]^ It has been proposed that people do not adopt an equally intentional stance toward robots, computers, and other artificial agents as compared to humans.^[^ ^35^ ^]^ In previous studies, the stronger activation in the mentalizing network has been repeatedly found when interacting with a human compared to a computer or a robot.^[^ ^36^ *^–^* ^40^ ^]^ Therefore, we hypothesize that NLP‐produced language results in distinct neural activity in the mentalizing network as compared to human‐produced language.

Previous evidence has shown that a person's language usage patterns are stable across context,^[^ ^17^ *^,^* ^19^ ^]^ whereas the NLP systems have not yet reached the capacity to produce language with stable language usage patterns.^[^ ^23^ ^]^ Previous studies have also reported that relatively stronger activation in the mentalizing network can be detected when a human participant is interacting with a human as compared to interacting with a computer or a robot,^[^ ^36^ *^–^* ^40^ ^]^ suggesting that NLP‐produced language may result in differential neural activity in the mentalizing network.

**Figure 1:**
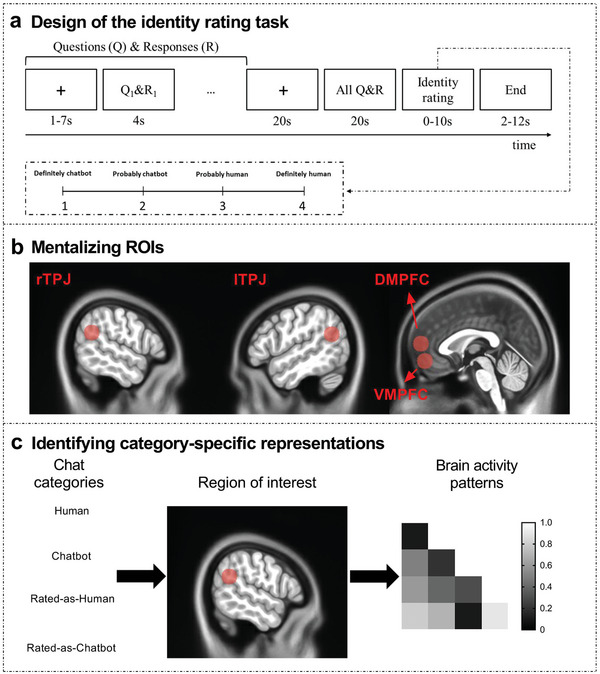
a) Design of the identity rating task. The judge was required to read a chat between Participant A and Participant B and then judge the identity of the Participant B after reading each chat (each interrogator examined 16 chats) (8 chatbot's and 8 human's). The identity rating judgment posed the following question: Is the Participant B a chatbot or a human being? (1‐ definitely chatbot; 2‐ probably chatbot; 3‐ probably human; 4‐ definitely human). b) Mentalizing regions of interest (ROIs). We defined a priori ROIs within the mentalizing network including the right temporoparietal junction (rTPJ), left temporoparietal junction (lTPJ), dorsomedial prefrontal cortex (DMPFC), and ventromedial prefrontal cortex (VMPFC). c) Identifying category‐specific representations. For each chat, we measured the activation value in ROIs to classify the chat categories.

In the present study, we addressed the hypothesis that the variance of personality perceived from NLP‐produced language is larger than that from human‐produced language. The NLP‐produced language was obtained from a social chatbot, Google Meena (in English).^[^ ^41^ *^,^* ^42^ ^]^ Twenty participants were recruited to evaluate the personalities of the respondent of each chat based on the Big Five Inventory.^[^ ^43^ ^]^ We calculated the variance of each of the five personality dimensions (i.e., openness, conscientiousness, extraversion, agreeableness, and neuroticism) for each chat and compared the variance between chat categories (i.e., human versus chatbot chats). To replicate the result, we also collected chatbot chats from another chatbot named as XiaoIce, a Chinese social chatbot designed by Microsoft. Then we used identity rating tasks (**Figure** **1**a) and functional magnetic resonance imaging (fMRI) to analyze the patterns of activity in the mentalizing network exposed to the subjectively indistinguishable chatbot and human chats. Twenty‐seven participants were evaluated, with each participant required to judge 8 human chats and 8 chatbot chats. According to the identity rating score, we screened out subjectively indistinguishable chatbot and human chats. We examined whether subjectively indistinguishable chatbot and human chats can be characterized by multi‐voxel fMRI activity patterns^[^ ^44^ ^]^ of mentalizing network regions, including the right temporoparietal junction (rTPJ), the left temporoparietal junction (lTPJ), the dorsomedial prefrontal cortex (DMPFC) and the ventromedial prefrontal cortex (VMPFC)^[^ ^45^ ^]^ (Figure 1b,c). Finally, we explored the reproducibility of classification results by performing a Chinese version of the identity rating task. Our behavioral results revealed that variance of personality perceived from chatbot chats generated by both Google Meena and Microsoft XiaoIce was larger than for human chats. Our neuroimaging data indicated distinct patterns of brain activity in the mentalizing network in response to chatbot versus human chats that cannot be distinguished subjectively. The present study supports that there is instability in the usage patterns of NLP‐produced language and this instability results in distinct patterns of mentalizing network activity between NLP‐produced and human‐produced language, illustrating adding a judge's implicit perception as an additional criterion is a promising empirical basis for measuring the quality of NLP‐produced language.

## 2 Results

### 2.1 Personality as Perceived from the Chatbot Chats Was not Stable

To test the possibility that personality perceived from the chatbot chats was not stable across context, 8 chatbot chats produced by Meena, an English social chatbot developed by Google, and 8 human chats produced by different individuals were obtained. All the chatbot and human chats were obtained in continuous, free conversation. The personalities of the respondent of each chat were evaluated by 20 participants, all of whose mother tongue or official language is English, based on the Big Five Inventory. We calculated the variance of each of the five personality dimensions (i.e., openness, conscientiousness, extraversion, agreeableness, and neuroticism) for each chat (8 human and 8 chatbot chats) and compared the variance between chat categories (i.e., human versus chatbot chats) using permutation tests. The results showed that the variance of the agreeableness dimension perceived from chatbot chats was larger than that of human chats (agreeableness: permuted *p* = 0.01).

To replicate the larger variance of perceived personality from chatbot chats, we also collected chats from another chatbot named as XiaoIce, a Chinese social chatbot designed by Microsoft. we collected 42 human chats and 42 chatbot chats and then recruited 165 independent participants to evaluate the personalities of the respondents for these chats based on the Big Five Inventory (the number of participants evaluating each chat ranged from 10 to 20, mean = 16.3); note that all of the participants for evaluation are native Chinese speakers. We calculated the variance of each personality dimension for each chat and compared the variance between chat categories (i.e., human and chatbot chats) using permutation tests. Briefly, the results showed that the variance of the neuroticism, agreeableness, conscientiousness, and openness dimensions perceived from chatbot chats was larger than that from human chats (neuroticism: permuted *p* < 0.001; agreeableness: permuted *p* < 0.001; conscientiousness: permuted *p* < 0.001; openness: permuted *p* = 0.009).

### 2.2 Behavioral Results of the Identity Rating Task

To determine whether chats from Google Meena and humans are characterized by distinct patterns of brain activity, we used an identity rating task (Figure 1a) with fMRI wherein each participant acted as a judge who was required to read a chat between “Participant A” and “Participant B”, and then judged the identity of the “Participant B” after reading each chat. Each volunteer defined as “Participant A” conversed 1:1 with the chatbot or a person (i.e., “Participant B”) without expectation or instructions about the content of the conversation to produce a chat. For each chat, a conversation round between “Participant A” and “Participant B” was presented for 4 s; this was done one by one, with a variable interval (1–7 s, mean = 4 s). After the presentation of all conversation rounds, the complete chat was presented following a 20 s interval. The identity rating judgment posed the following question: is the “Participant B” in this chat a chatbot or a human being? (1‐ definitely chatbot; 2‐ probably chatbot; 3‐ probably human; 4‐ definitely human). Twenty‐seven participants were involved, and each participant was required to judge 16 chats (8 human chats and 8 chatbot chats). Our results indicated that there were 9.41 ± 1.80 chats rated as “Human” (identity rating score ≥ 3). Among them, there were 4.70 ± 1.56 human chats rated as “Human” (HRH) and 4.70 ± 1.27 chatbot chats rated as “Human” (CRH). There were 6.59 ± 1.80 chats rated as “Chatbot” (identity rating score ≤ 2). Among them, there were 3.30 ± 1.56 human chats rated as “Chatbot” (HRC) and 3.30 ± 1.27 chatbot chats rated as “Chatbot” (CRC). We also collected participants’ Sensibleness and Specificity Average (SSA),^[^ ^42^ ^]^ which is a metric combining two fundamental aspects of humanlike chatbots: making sense and being specific. Our result indicated a significant positive correlation between identify rating score and Sensibleness and Specificity Average (SSA) (*r* = 0.669, *p* < 0.005; Figure S1, Supporting Information).

### 2.3 Classifying Chat Identities from Mentalizing Network Region Patterns

We examined whether chats from Google Meena and humans are characterized by the multi‐voxel fMRI activity patterns of mentalizing network regions, including the rTPJ, lTPJ, DMPFC and VMPFC. We defined a priori regions of interest (ROIs) according to a previous meta‐analysis study of the mentalizing network (coordinate in Montreal Neurological Institute [MNI] space; spheres of 10 mm radius; rTPJ: *x* = 56, *y* = −54, *z* = 26; lTPJ: *x* = −52, *y* = −58, *z* = 24; DMPFC: *x* = 0, *y* = 58, *z* = 12; VMPFC: *x* = 0, *y* = 52, *z* = −8; Figure 1b).^[^ ^45^ ^]^ The analyses focused on the activity patterns upon reading chats (i.e., from the first question and its response to the last question and its response for a given chat). The activation value of each chat was assessed by a general linear model (GLM) and used for multi‐voxel pattern analysis (MVPA; Figure 1c). A permutation test was used to assess the significance of the results.

**Figure 2:**
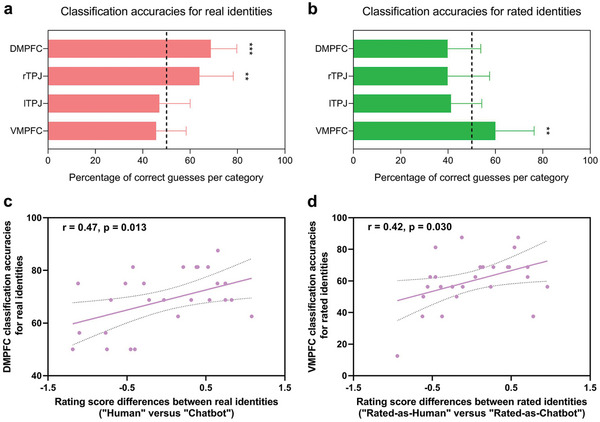
a) Classification accuracies for real identities (human versus chatbot). b) Classification accuracies for rated identities (rated‐as‐human versus rated‐as‐chatbot). c) Correlation between the DMPFC classification accuracies for real identities and identity rating score differences between real identities. d) Correlation between the VMPFC classification accuracies for rated identities and identity rating score differences between rated identities. Dashed line represents chance level (50%); * *p* < 0.05, ** *p* < 0.01, and *** *p* < 0.001; Error bars indicate the s.e.m.

We first determined whether the activity patterns could classify real identities between the chat categories (i.e., human versus chatbot). The mean classification accuracy for real identities was above the permutation‐based significance level for the DMPFC (chance level = 50%, mean classification accuracy = 68.75% ± 10.96%, permuted *p* = 0.0002; **Figure** **2**a) and the rTPJ (mean classification accuracy = 63.89% ± 14.33%, permuted *p* = 0.0012), but not for the lTPJ (mean classification accuracy = 46.99% ± 13.01%, permuted *p* = 0.90) or the VMPFC (mean classification accuracy = 45.60% ± 12.72%, permuted *p* = 0.97).

We next determined whether the activity patterns could classify rated identities between the chat categories (i.e., rated‐as‐human versus rated‐as‐chatbot). The mean classification accuracy for rated identities was above the permutation‐based significance level for the VMPFC (mean classification accuracy = 59.95% ± 16.38%, permuted *p* = 0.0030; Figure 2b), but not for the DMPFC (mean classification accuracy = 39.81% ± 14.00%, permuted *p* = 1), the rTPJ (mean classification accuracy = 39.81% ± 17.69%, permuted *p* = 1) or the lTPJ (mean classification accuracy = 41.20% ± 13.00%, permuted *p* = 1).

Correlation analyses indicated that larger identity rating score differences between real identities were associated with higher DMPFC classification accuracies for real identities (*r* = 0.47, *p* = 0.013; Figure 2c); larger identity rating score differences between rated identities were associated with higher VMPFC classification accuracies for rated identities (*r* = 0.42, *p* = 0.030; Figure 2d).

### 2.4 Classifying Chat Identities between Subjectively Indistinguishable Chatbot and Human Chats from Activity Patterns of the DMPFC and rTPJ

**Figure 3:**
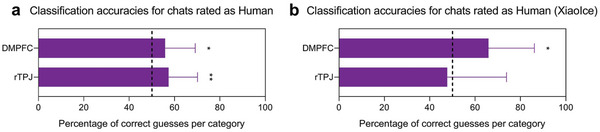
a) Classification accuracies for chats rated as “Human.” The activity patterns of the DMPFC and rTPJ could still classify real identities between subjectively indistinguishable human and chatbot chats. b) Classification accuracies for chats rated as “Human” in the replicated experiment. Chatbot chats were produced by XiaoIce, a Chinese social chatbot designed by Microsoft. Dashed line represents chance level (50%); * *p* < 0.05, **p < 0.01, and ***p < 0.001; Error bars indicate the s.e.m.

Our results indicate that the activity patterns of the DMPFC and the rTPJ can be used to informatively classify the real identities of chats (i.e., human versus chatbot). Some may argue that the classification accuracies were mainly contributed by subjectively distinguishable human and chatbot chats (i.e., HRH versus CRC), rather than by subjectively indistinguishable human and chatbot chats (i.e., HRH versus CRH). We, therefore, examined whether the activity patterns of the DMPFC and rTPJ could still be used to classify real identities between subjectively indistinguishable human and chatbot chats (i.e., HRH versus CRH). The mean classification accuracy for real identities between subjectively indistinguishable human and chatbot chats was still above the permutation‐based significance level for the DMPFC (mean classification accuracy = 55.88% ± 13.23%, permuted *p* = 0.021; **Figure** **3**a) and the rTPJ (mean classification accuracy = 57.34% ± 12.77%, permuted *p* = 0.0032).

### 2.5 Across‐Subject Similarity Correlation

**Figure 4:**
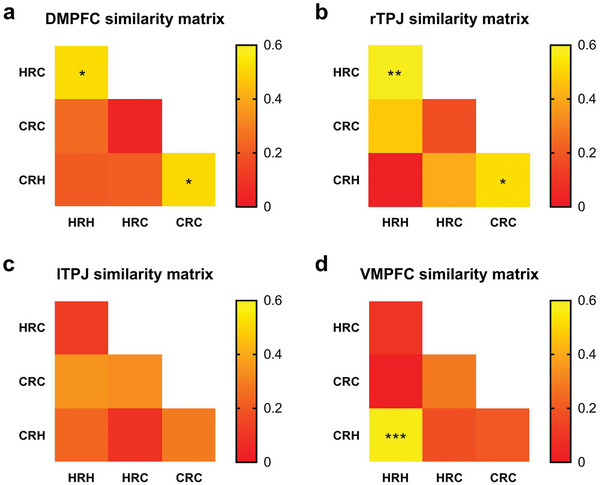
a) DMPFC Similarity matrix. b) Right TPJ Similarity matrix. c) Left TPJ Similarity matrix. d) VMPFC Similarity matrix. HRH = human chat rated as “Human”, HRC = human chat rated as “Chatbot”, CRH = chatbot chat rated as “Human”, and CRC = chatbot chat rated as “Chatbot.” * *p* < 0.05, **p < 0.01, and ***p < 0.001.

We next explored the activity similarity between the four chat categories (i.e., HRH, HRC, CRH, and CRC) by using an across‐subject correlation. The purpose of this analysis was to determine whether specific pairs of chat categories reveal similar activity across subjects. We averaged the activation value for each chat category and extracted the average activation value of each ROI. We calculated the similarity matrices within ROIs by calculating the correlation between all pairs of chat categories across subjects. The DMPFC similarity matrix indicated that there was similar activity between HRC and HRH (*r* = 0.52, FDR‐corrected *p* = 0.034; **Figure** **4**a) and between CRH and CRC (*r* = 0.51, FDR‐corrected *p* = 0.035). The rTPJ similarity matrix indicated that there also was similar activity between HRC and HRH (*r* = 0.59, FDR‐corrected *p* = 0.0066; Figure 4b) and between CRH and CRC (*r* = 0.52, FDR‐corrected *p* = 0.027). These results indicate that chats produced by a specific generator (human or chatbot) elicit similar activities in the DMPFC and rTPJ across subjects, regardless of the identity judgments. No significant differences were detected in the lTPJ matrix (Figure 4c). The VMPFC similarity matrix indicated that there was similar activity between CRH and HRH (*r* = 0.66, FDR‐corrected *p* = 0.0006; Figure 4d), indicating that chats rated as “Human” elicited similar activity in the VMPFC across subjects, regardless of the real identities.

### 2.6 Whole‐Brain Classification Searchlight Analysis

**Figure 5:**
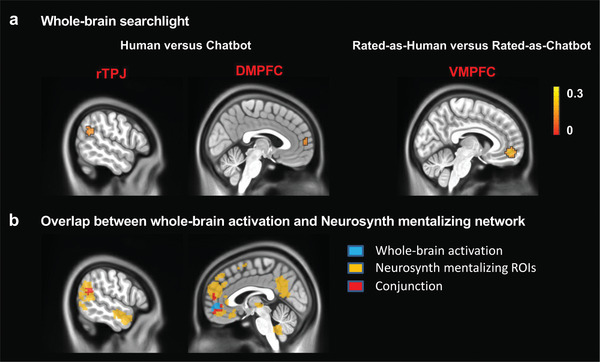
a) Whole‐brain searchlight results. For the real identity classification, pattern classification was robust in the rTPJ and DMPFC. For the rated identity classification, pattern classification was robust in the VMPFC. b) Overlap between whole‐brain activation and Neurosynth mentalizing network. rTPJ: right temporoparietal junction; DMPFC: dorsomedial prefrontal cortex; VMPFC: ventromedial prefrontal cortex.

We performed an exploratory whole‐brain searchlight analysis to identify brain regions encoding category‐specific information in each identity classification (i.e., real and rated identity classifications) separately across the whole brain. For the real identity classification, pattern classification was robust in the rTPJ and the DMPFC (a cluster‐level correction with threshold of family‐wise error [FWE] correction with *p* < 0.05 using an initial cluster‐forming threshold of *p* < 0.001 [uncorrected]; **Figure** **5**a). For the rated identity classification, pattern classification was robust in the VMPFC (Figure 5a). To explore the relationship between whole‐brain activation and the mentalizing network, we searched for the term “mentalizing” and extracted the corresponding activation masks using the platform Neurosynth (neurosynth.org), and performed conjunction analysis between whole‐brain activation and Neurosynth mentalizing masks. Our results indicated an overlap between the whole‐brain activation and the mask generated from NeuroSynth “mentalizing” meta‐analysis (Figure 5b).

### 2.7 Results Found in the Chinese Version of the Identity Rating Task

We explored the reproducibility of classification results by performing a Chinese version of the identity rating task. In the Chinese version of the identity rating task, 2 chatbot chats were produced by XiaoIce, a Chinese social chatbot designed by Microsoft, and 6 human chats were produced by different individuals. We then recruited 49 participants, whose mother tongue is Chinese, to perform the Chinese version of the identity rating task. The experiment followed the same procedures as detailed above for the English version of the identity rating task. Our results indicated that there were 5.51 ± 1.62 chats rated as “Human” (identity rating score ≥ 3). There were 2.49 ± 1.62 chats rated as “Chatbot” (identity rating score ≤ 2).

We first determined whether the activity patterns could classify real identities between the chat categories (i.e., human versus chatbot). The mean classification accuracy for real identities was above the permutation‐based significance level for the DMPFC (chance level = 50%, mean classification accuracy = 57.14% ± 18.40%, permuted *p* = 0.0068; Figure S2a, Supporting Information) and the lTPJ (mean classification accuracy = 60.20% ± 19.71%, permuted *p* = 0.0008), but not for the rTPJ (mean classification accuracy = 47.96% ± 24.91%, permuted *p* = 0.69) or the VMPFC (mean classification accuracy = 47.45% ± 24.60%, permuted *p* = 0.75).

We next determined whether the activity patterns could be used to classify rated identities between the chat categories (i.e., rated‐as‐human versus rated‐as‐chatbot). The mean classification accuracy for rated identities was above the permutation‐based significance level for the VMPFC (mean classification accuracy = 56.64% ± 14.71%, permuted *p* = 0.0048; Figure S2b, Supporting Information) and the lTPJ (mean classification accuracy = 55.29% ± 16.77%, permuted *p* = 0.016), but not for the DMPFC (mean classification accuracy = 48.42% ± 20.80%, permuted *p* = 0.69) or the rTPJ (mean classification accuracy = 40.99% ± 23.11%, permuted *p* = 1). Note that correlation analyses indicated no significant correlations between classification accuracies and identity rating score differences for any ROIs.

We then determined whether the activity patterns of the DMPFC and rTPJ could still be used to classify real identities between subjectively indistinguishable human and chatbot chats (i.e., HRH versus CRH). The mean classification accuracy for real identities between subjectively indistinguishable human and chatbot chats was still above the permutation‐based significance level for the DMPFC (mean classification accuracy = 65.91% ± 20.23%, permuted *p* = 0.027; Figure 3b) but not for the rTPJ (mean classification accuracy = 47.73% ± 26.11%, permuted *p* = 0.73). Owing to the limited number of chatbot chats, we did not perform across‐subject similarity correlation analysis.

We performed the same exploratory whole‐brain searchlight analysis as described above for the English version of the identity rating task to identify brain regions encoding category‐specific information in each identity classification (i.e., real and rated identity classifications) separately across the whole brain. Pattern classification was robust in the left precentral gyrus (a cluster‐level correction with threshold of family‐wise error [FWE] correction with *p* < 0.05 using an initial cluster‐forming threshold of *p* < 0.001 [uncorrected]) for the real identity classification. For the rated identity classification, pattern classification was robust in no regions.

### 2.8 A Separate Reading Task Excludes Chat Content per se from the Observed Classification

We used a reading task to test whether chat category‐specific classification in the mentalizing network occurs only for the situation where participants are required to infer the identity of Participant B from a chat's content. The experimental procedure of the reading task was almost the same as that of the Chinese version of the identity rating task (Figure S3, Supporting Information); the difference is that for the reading task the participants answered one multiple‐choice question about the details related to a given chat. We later asked the participants to give an identity rating score for the chat “Participant B”, but they were not informed about this requirement in advance. Forty‐six participants were assessed with the reading task.

The mean classification accuracy for real identities was above the permutation‐based significance level for the rTPJ (chance level = 50%, mean classification accuracy = 56.52% ± 20.71%, permuted *p* = 0.021; Figure S4a, Supporting Information) but not for the DMPFC (mean classification accuracy = 53.26% ± 23.34%, permuted *p* = 0.20), the lTPJ (mean classification accuracy = 48.91% ± 21.71%, permuted *p* = 0.71), or the VMPFC (mean classification accuracy = 49.46% ± 18.63%, permuted *p* = 0.64). The mean classification accuracy for rated identities was not above the permutation‐based significance level for any ROIs (DMPFC: mean classification accuracy = 54.73% ± 24.28%, permuted *p* = 0.14; rTPJ: mean classification accuracy = 48.42% ± 23.04%, permuted *p* = 0.69; lTPJ: mean classification accuracy = 52.59% ± 21.59%, permuted *p* = 0.27; VMPFC: mean classification accuracy = 51.58% ± 24.08%, permuted *p* = 0.34; Figure S4b, Supporting Information). The mean classification accuracy for real identities between subjectively indistinguishable human and chatbot chats was above the permutation‐based significance level for no regions (DMPFC: mean classification accuracy = 48.61% ± 22.53%, permuted *p* = 0.65; rTPJ: mean classification accuracy = 51.62% ± 21.38%, permuted *p* = 0.39).

These findings from the reading task assessing a group of identity‐judgment naïve participants empirically support that the chat category‐specific classification in the mentalizing network occurs only when participants are required to judge the identity of the “Participant B” based on a chat's content.

## 3 Discussion

Our behavioral result showed a larger variance of personality perceived from chatbot chats compared to human chats. Neuroimaging analyses revealed that subjectively indistinguishable chatbot and human chats elicited distinct patterns of brain activity in the mentalizing network regions including the DMPFC and rTPJ. Chats produced by a specific generator (human or chatbot) elicited similar activity in the DMPFC and rTPJ across subjects regardless of the identity judgments.

Speaker meaning is the intended meaning a speaker wants to communicate with a specific utterance.^[^ ^46^ ^]^ The comprehensions of the speaker's meaning are more than the retrieval of word meaning or sentence‐level,^[^ ^47^ ^]^ which requires the mentalizing network.^[^ ^48^ ^]^ Mentalizing describes the cognitive activities through which we infer the inner thoughts and intentions of others. To judge the identity of “Participant B” in the identity rating task, the participant needs to retrieve language and then infer both meaning and social information. Mentalizing establishes the context that enables insight into the identity of the mind of “Participant B”, leading to a better understanding of the respondent. Early investigations into the neural basis of mentalizing observed a typical network of activation, which included the medial prefrontal cortex and TPJ.^[^ ^49^ *^,^* ^50^ ^]^ Various mentalizing tasks consistently activate the medial prefrontal cortex and TPJ.^[^ ^51^ *^,^* ^52^ ^]^ The right TPJ is implicated in mental‐state reasoning, that is, thinking about other people's beliefs, emotions and desires. Activation in the right TPJ has been also shown to associate with autistic spectrum disorder syndrome severity in a self‐other mental‐state reasoning task.^[^ ^53^ ^]^ The DMPFC has been implicated in a process known as continuous internal monitoring.^[^ ^54^ ^]^ Conflict monitoring and error monitoring are also thought to depend on some underlying processes instantiated in the DMPFC. We interpret our results to indicate that, for the subjectively indistinguishable chatbot chats, participants may continuously evaluate the unapparent mismatches between chatbot and human by inferring interacting partner's beliefs, emotions and desires in the mentalizing network. Our neuroimaging results demonstrate that neural activity in the mentalizing network can be used to distinguish NLP‐produced language from human‐produced language, even when these cannot be distinguished subjectively.

It is possible that the neural activity patterns we detected in the mentalizing network could be due to the change in usage patterns of NLP. Recall our initial findings showing that chatbot chats exhibit a larger variance of perceived personality compared to human chats (in dimensions including agreeableness and neuroticism). To our understanding, personality traits represent a reliable and stable way of indexing an individual's thinking, feeling, and behavior. Hundreds of studies using different methods, instruments, and populations have consistently found high levels of retest stability for personality traits over intervals of up to 40 years.^[^ ^55^ ^]^ A meta‐analysis^[^ ^56^ ^]^ of 243 retest coefficients for personality traits showed that the average observed value after an interval of 15 years would be ≈0.60 (correlations more than 0.50 are large based on Cohen's rule of thumb^[^ ^57^ ^]^). Using writing samples of college students, researchers found reliable effects for Big Five personality dimensions on word choice.^[^ ^20^ ^]^ It also bears emphasis that the previous work has established that people's language usage patterns satisfy the basic psychometric requirements of consistency across context and stability across time.^[^ ^20^ *^,^* ^58^ *^,^* ^59^ ^]^ Therefore, the personality perceived from the language materials of a given person should be stable. However, recall that current NLP methods are based on the modeling of language from very large datasets comprising language materials produced by many different people. As a consequence, NLP‐produced language inherently represents multiple personalities, suggesting that the language usage patterns of NLP are not stable and not consistent across context. Our data support that the participants did not subjectively detect any aberrant changes in some but not all of the NLP usage patterns. However, we show that this aberrant change was reliably detectable based on neural activity in the mentalizing network.

Our results support that adding neural activity as an additional criterion would produce more precise and comprehensive information when measuring the quality of NLP‐produced language. We show that subjectively undetectably subtle differences between NLP‐produced and human‐produced language are detected in the mentalizing network; iterative testing and incorporation of experimental insights could dramatically advance progress in the development of NLP and perhaps other fields of artificial intelligence. In psychology, a self‐report is any measure or survey that relies on an individual's report of his/her behaviors, attitudes, beliefs, or symptoms. It can yield much valuable information. Most experts in psychological research and diagnosis suggest that self‐report should not be used alone because of its limitations.^[^ ^60^ ^]^ Measures are best done when combining self‐report with other information, such as an individual's physiological or neural data. This “multi‐method” assessment might provide more accurate data of the subject.

Our results revealed that a separate circuit in the mentalizing network is responsible for processing subjectively rated identities between chatbot and human. Our results found that the activity patterns could be used to classify rated identities between the chat categories (i.e., rated‐as‐human versus rated‐as‐chatbot). Furthermore, chats rated as “Human” elicited similar activity in the VMPFC across subjects. That is to say, regardless of the real identity (chatbot or human) of “Participant B”, chats identified as human‐produced induced similar activity in the VMPFC. The VMPFC has been implicated in self‐referential judgments,^[^ ^61^ *^–^* ^63^ ^]^ pointing to a link between thinking about oneself and thinking about others. Judging a chat as human‐produced might prompt the judge to make inferences about the human's characteristics, beliefs and/or mental state, which might trigger self‐referential processing as mediated by the VMPFC.

## 4 Conclusion

Identification of informative criteria for measuring NLP‐produced language quality can support the development of ever‐better NLP tools. The present study provides empirical evidence that the usage patterns of NLP‐produced language are not stable across different subjects of conversations and shows that this instability results in distinct patterns of mentalizing network activity between NLP‐produced and human‐produced language. Our study contributes to the field of NLP by showing that endowing a dialogue system with particular personality traits is essential to deliver more human‐like conversations. Another contribution is that our study successfully applies neuroimaging technology to the field of artificial intelligence for assessing the NLP‐produced language quality. Future studies might explore the feasibility of adding neural activity data as an implicit index to the assessment criteria for standard Turing tests. Besides, the relationship between the judge's language ability and neural activity sensitivity should be clarified in the future with the large sample size and the real‐time communication just like standard Turing tests.

In the identity rating task, participants read the chat contents and gave an identity rating score for the chat interlocutor, and this did allow the classification of subjectively indistinguishable chatbot chats and human chats. Provided access to sufficiently sensitive neuroimaging instruments can be obtained, it would be fascinating to determine if our findings about implicit perception differences can be replicated in a standard Turing test. We anticipate that adding a judge's implicit perception data could become a standard criterion for conducting Turing tests.

Concerning study limitations, the sample size for our fMRI analysis was small, in part owing to limited access and long set‐up times for fMRI technology. Naturally, this limits the statistical power of our analyses; ideally, a cheap‐to‐produce and easy‐to‐use, yet suitably sensitive apparatus for monitoring the relevant brain regions (e.g., DMPFC and rTPJ) would substantially expand the use of neuroimaging and implicit perception differences for assessing NLP‐produced language. Another limitation was the somewhat homogenous population of humans used to generate the human chat content for the second of the two identity rating tasks we examined in this study: high‐school girls, aged 15–19. This was expanded in the first experiment (based on human chat content from randomly selected humans), but there is still a gap in the scope of the materials used to train the examined XiaoIce and Meena versus the scope of the human materials. Thus, caution is warranted regarding any interpretations around topics beyond simply distinguishing machine versus human (i.e., for any personality or language use investigations).

## 5 Experimental Section

### Participants

Thirty‐six participants whose mother tongue or official language is English (such as Canadian, Nigerian, Pakistani, etc.) were recruited to complete the identity rating task. Data from 6 participants were excluded because of excessive head motion (for all participants, any time repetition (TR) with motion exceeding 0.3 mm was censored; participants who had more than 15% of their TRs censored were excluded from further analysis), data from 3 participants were excluded because they failed to complete the task, yielding an effective sample size of 27 participants (5 females; age = 29.9 ± 2.9 years). Fifty‐five participants were recruited to perform the Chinese version of the identity rating task. Among them, 2 participants were excluded due to equipment malfunction; 4 participants were excluded due to excessive head motion during fMRI. The final sample comprised 49 participants (age: 21.3 ± 2.5 years; 24 females). Forty‐nine participants were recruited to complete the reading task. Data from 3 participants were excluded because of excessive head motion, yielding an effective sample size of 46 participants (36 females; age = 21.5 ± 2.2 years). The sample size was determined using a medium effect size (Cohen's *d* = 0.5). Doing a power analysis using G*power suggested a sample size of 27 for 0.8 power. The study was approved by the Research Ethics Committee of the University of Science and Technology of China (NO. 2020‐N(H)‐099), and written informed consent was obtained from all participants, consistent with the Declaration of Helsinki. The methods were carried out in accordance with the approved guidelines.

### Chat Materials

In the identity rating task, chatbot and human chats were provided by the Brain Team of Google Research^[^ ^42^ ^]^ and are available at https://www.github.com/google‐research/google‐research/tree/master/meena/. Volunteers collectively had conversations with Meena (i.e., “chatbot chat” defined in the study), an English social chatbot designed by Google, and conversations with randomly selected human respondents (i.e., “human chat” defined in the study). Volunteers conversed (by text) 1:1 with Meena or a person; the content of the conversations was varied, including for example topics like hobbies, weather and sports, among others. During the collection of chat materials, the content of the conversation was unlimited and could be changed freely. A conversation was required to last at least 7 rounds (with the longest lasting 14 rounds). Eight conversations with Meena and 8 conversations with humans which had similar numbers of conversation rounds (range: 8–10; mean: chatbot = 8.75, human = 8.75, *p* = 1) and total words (range: 148–221; mean: chatbot = 180.50, human = 182.25, *p* = 0.88) were chosen.

In the Chinese version of the identity rating task, 2 chatbot chats were produced by XiaoIce, a Chinese social chatbot designed by Microsoft, and 6 human chats were produced by different persons. The details of the assessment of chat materials are shown in the Supporting Information: production process of chat materials for the Chinese version of the identity rating task.

### Personality Perception of Chat Materials

The personalities of the “Participant B” for each of the 16 chat materials (i.e., 8 human chats and 8 chatbot [i.e., Meena] chats) were evaluated by 20 participants according to the Big Five Inventory (see the details in Supporting Information). To test the hypothesis that the variance of personality perceived from chatbot chats was larger than that from human chats, the variance of each of the five dimensions for each chat (8 human and 8 chatbot [i.e., Meena] chats) was calculated and the variance between chat categories using permutation tests was compared.

### Identity Rating Task

The identity rating task consisted of four fMRI runs, each run comprising four chats. Participants were required to judge 16 chats (8 human chats and 8 chatbot chats) and informed that each chat contains several conversation rounds between “Participant A” and “Participant B”; the “Participant A” in each chat was a human and the “Participant B” was either a human or a chatbot, and they needed to judge the identity of “Participant B” based on the content of each chat. In the identity rating task, a conversation round between “Participant A” and “Participant B” was presented in the center of the screen to a participant for 4 s; this was done one by one, with a variable interval (1–7 s, mean = 4 s). After each conversation round in a chat was presented, all conversation rounds were presented simultaneously following a 20 s interval. Subsequently, the identity rating judgment posed the following question: is “Participant B” in this chat a chatbot or a human being? (1‐ definitely chatbot; 2‐ probably chatbot; 3‐ probably human; 4‐ definitely human). To visualize the presentation of the identity rating task, a video demo is available at https://rec.ustc.edu.cn/share/85b13e20‐dd9e‐11ec‐ade7‐0d670f3af599.

In the Chinese version of the identity rating task, each fMRI run comprised two chats. The experiment followed the same procedures above.

### Reading Task

The experimental procedure of the reading task was almost the same as that of the Chinese version of the identity rating task (Figure S3, Supporting Information); the difference was that for the reading task the participants did not need to report an identity rating score about the chat “Participant B.” Rather, the participants answered one multiple‐choice question about the details related to a given chat. For example, participants were required to answer: what does the “Participant B” feel happy about? 1‐ staying at home; 2‐ traveling; 3‐ falling in love; 4‐ sleeping. The goal here was to encourage the participants to focus on the chat. The authors later asked the participants to give an identity rating score for the chat “Participant B”, but they were not informed about this requirement in advance.

### fMRI Data Acquisition

Gradient echo‐planar imaging data were acquired using a 3.0 T GE discovery MR750 with a circularly polarized head coil, at the Medical Image Center, University of Science and Technology of China. A T2*‐weighted echo‐planar imaging sequence (FOV = 240 mm, TE = 30 ms, TR = 2000 ms, flip angle = 85°, matrix = 64 × 64) with 33 axial slices (no gaps, voxel size: 3.75 × 3.75 × 3.7 mm^3^) covering the whole brain was used to acquire the functional MR images. High‐resolution T1‐weighted spin‐echo imaging data were also acquired for anatomical overlays and 3D gradient echo‐planar imaging for stereotaxic transformations after functional scanning. Before entering the scanner, participants were instructed to keep their heads still during all scans. During the identity rating task, four functional scan runs occurred, with each lasting 512 s. During the Chinese version of the identity rating task, four functional scan runs occurred, with each lasting 424 s.

### fMRI Data Preprocessing and Regression

Functional data were realigned to the lowest motion volume. The realigned images were normalized to the MNI standard space template with a resolution of 2 mm^3^. Raw data were corrected for temporal shifts between slices and for motion, and were temporally normalized (for each voxel, the signal of each volume was divided by the temporally averaged signal). Time series preprocessing was done using AFNI software. A general linear model (GLM) was used to identify neural responses exhibiting correlations with each chat material. In each chat, the regressors were as below: first reading stage: the period when a conversation round was presented one‐by‐one; second reading stage: the period when all conversation round were presented at the same time; and interval: the period between the first and second reading stages. These regressors were convolved with a hemodynamic response function (HRF) and simultaneously regressed against the blood oxygenation level‐dependent (BOLD) signal in each voxel. Six regressors for the head motion were also included. The *β* values for each chat's first reading stage were used for MVPA.

### MVPA

The authors used MVPA^[^ ^44^ ^]^ to test whether patterns of brain activity (*β* values for each chat's first reading stage) could classify the identities of chats. For the real identity classification, chats produced by volunteers were labeled as “Human”, and chats produced by chatbot were labeled as “Chatbot.” For the rated identity classification, chats with an identity rating score ≥ 3 were labeled as “Rated‐as‐Human”, and chats with an identity rating score ≤ 2 were labeled as “Rated‐as‐Chatbot.” The discriminability of patterns for the two categories within‐subject in each identity classification (real and rated identity classifications) was tested separately with CoSMoMVPA^[^ ^44^ ^]^ using the linear support vector machine (SVM) tool. Classification training and testing were done using a leave‐one‐chunk‐out cross‐validation strategy.^[^ ^44^ ^]^

Due to the unbalanced number of human and chatbot chats in the Chinese version of the identity rating task, the *β* values for the chat's first reading stage to balance the number of each chat category before the MVPA were averaged. Due to the limited number of chatbot chats in the Chinese version of the identity rating task, data from some participants did not meet the minimum number of all chat categories for the MVPA. Finally, data from 37 of 49 participants were assessed for the rated identity classification; data from 11 of 49 participants were assessed for classifying the real identities between subjectively indistinguishable human and chatbot chats. A similar situation occurred in the reading task. Data from 37 of 46 participants were assessed for the rated identity classification; data from 10 of 46 participants were assessed for classifying the real identities between subjectively indistinguishable human and chatbot chats.

### ROI Analysis

4 ROIs that were defined in a previous meta‐analysis study of the mentalizing network were here used. The coordinates of these ROIs calculated from a meta‐analysis were based on the “story type” of ToM task (coordinate in MNI space; spheres of 10 mm radius; rTPJ: *x* = 56, *y* = −54, *z* = 26; left TPJ: *x* = −52, *y* = −58, *z* = 24; DMPFC: *x* = 0, *y* = 58, *z* = 12; VMPFC: *x* = 0, *y* = 52, *z* = −8).^[^ ^45^ ^]^ The *β* value of each ROI was extracted and was applied to MVPA. Statistical significance of the classification accuracies for ROIs was determined using a permutation test that generates a set of 5000 chance classification accuracies by permuting the labels each time.

### Classification Searchlight

In an exploratory whole‐brain analysis, a searchlight analysis to identify brain regions coding category‐specific information in each identity classification (real and rated identity classifications) separately across the whole brain was performed. The radius of each sphere was adjusted such that each searchlight contained ≈100 voxels. For the correction of multiple comparisons, a cluster‐level correction with a threshold of family‐wise error (FWE) correction with *p* < 0.05 using an initial cluster‐forming threshold of *p* < 0.001 (uncorrected) was employed. To explore the relationship between whole‐brain activation and mentalizing network, the term “mentalizing” was searched for and the corresponding activation masks based on all 151 studies using the platform Neurosynth (neurosynth.org) was extracted, and the conjunction between whole‐brain activation and Neurosynth mentalizing masks was performed.

### Across‐Subject Similarity Correlation

The activity similarity between the four chat categories (i.e., HRH, HRC, CRH, and CRC) was explored by using an across‐subject correlation. The activation value for each chat category was averaged and the average activation value of each ROI was extracted. The similarity matrices within ROIs by calculating the correlation between all pairs of chat categories across subjects were calculated.

### Statistical Analysis

The variance between chat categories was compared by using permutation tests. The significance of the classification accuracies by MVPA was assessed by using permutation tests. The average classification accuracies were present as the format of mean ± standard deviation. Sample size (*n*) was calculated using G*power. Statistical analysis of data was analyzed by AFNI software, CoSMoMVPA, MATLAB, and SPSS.

## Conflict of Interest

The authors declare no conflict of interest.

## Author Contributions

Z.W. and Y.C. contributed equally to this work. Z.D.W., Y.C., L.X.Z., and X.C.Z. conceived and designed the study. Z.D.W. and Y.C. obtained the findings. Y.C., Q.Z., and P.Y.Z. were responsible for the acquisition of data. Z.D.W. and Y.C. analyzed and interpreted the data. P.Y.Z., Q.Z., Y.P., J.C.R., S.P.W., J.L., R.C.K., X.X., B.S.Q. and D.R.Z. provided administrative, technical or material support. X.C.Z. supervised the study. Z.D.W. and Y.C. drafted the paper and all authors contributed to the critical revision of intellectual content.

## Supporting information

Supporting Information

Supplemental Video 1

## Data Availability

The data that support the findings of this study are openly available in rec at https://www.rec.ustc.edu.cn/share/85b13e20‐dd9e‐11ec‐ade7‐0d670f3af599, reference number 7745.
