# Supplementary material for: Implicit Perception of Differences between NLP‐Produced and Human‐Produced Language in the Mentalizing Network
Source: Adv Sci (Weinh). 2023 Feb 7;10(12):2203990. doi: 10.1002/advs.202203990 (PMC10131862; doi:10.1002/advs.202203990)
Supplement: Supplementary file 1 — Supporting Information [file ADVS-10-2203990-s002.pdf]

ADVANCED SCIENCE
Open Access

Supporting Information

for *Adv. Sci.*, DOI 10.1002/advs.202203990

Implicit Perception of Differences between NLP-Produced and Human-Produced Language in the Mentalizing Network

*Zhengde Wei, Ying Chen, Qian Zhao, Pengyu Zhang, Longxi Zhou, Jiecheng Ren, Yi Piao, Bensheng Qiu, Xing Xie, Suiping Wang, Jia Liu, Daren Zhang, Roi Cohen Kadosh and Xiaochu Zhang**

Supplementary materials and methods

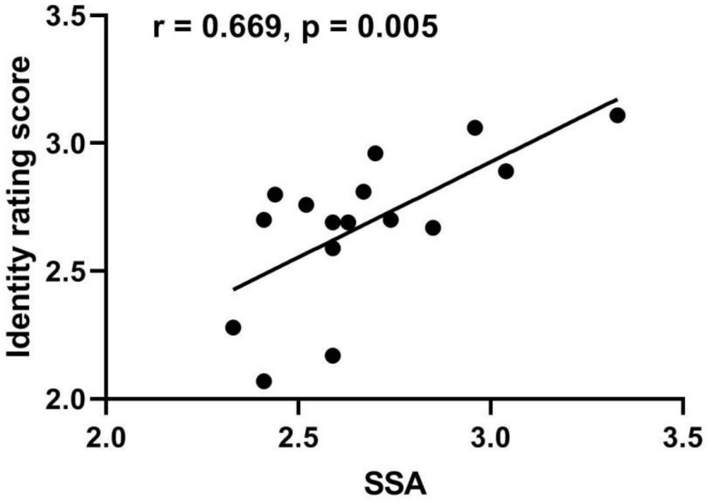

Supplementary Figure 1. *Relationship between identity rating score and Sensibleness and Specificity Average (SSA). A Pearson correlation analysis was used and a positive correlation between identify rating score and SSA was found (r = 0.669, p = 0.005). Each point is a different chat.*

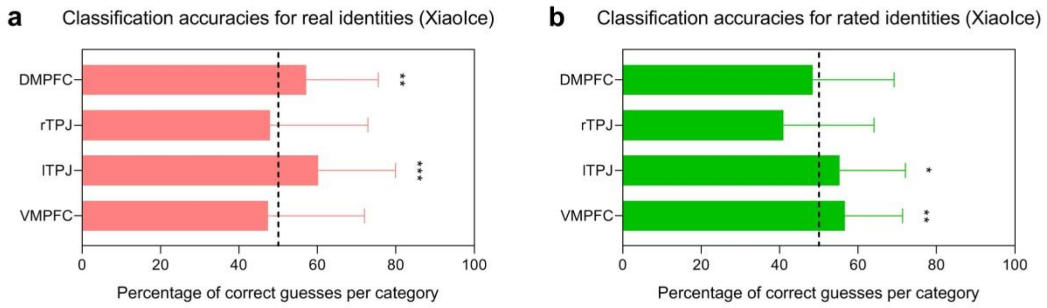

Supplementary Figure 2. (a) *Classification accuracies for real identities in the replicated experiment. Chatbot chats were produced by XiaoIce, a Chinese social chatbot designed by Microsoft.* ***(b)*** *Classification accuracies for rated identities in the replicated experiment. Dashed line represents chance level (50%); * p < 0.05, **p < 0.01 and ***p < 0.001; Error bars indicate the s.e.m.*

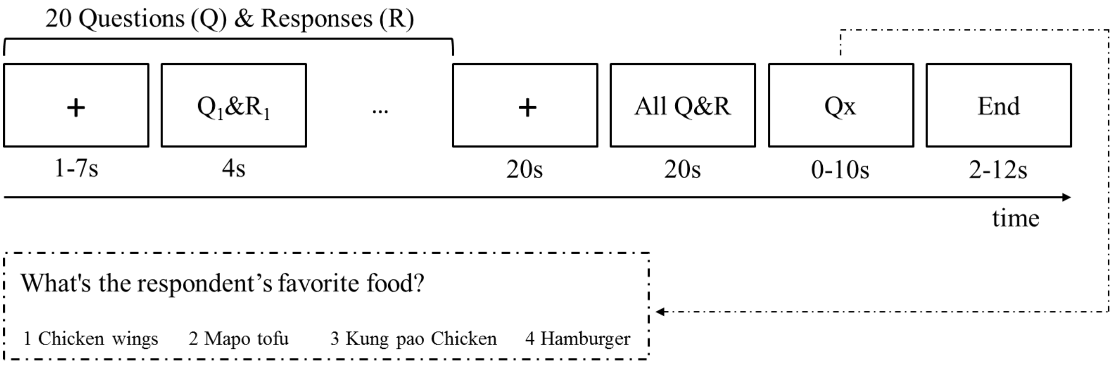

Supplementary Figure 3. *Reading task. Participants were required to read a chat and then answer a randomly selected question after reading each chat (each participant read 8 chats and answered 8 questions). Q: question; R: response;* Q_x*: a randomly selected question from the 20 questions.*

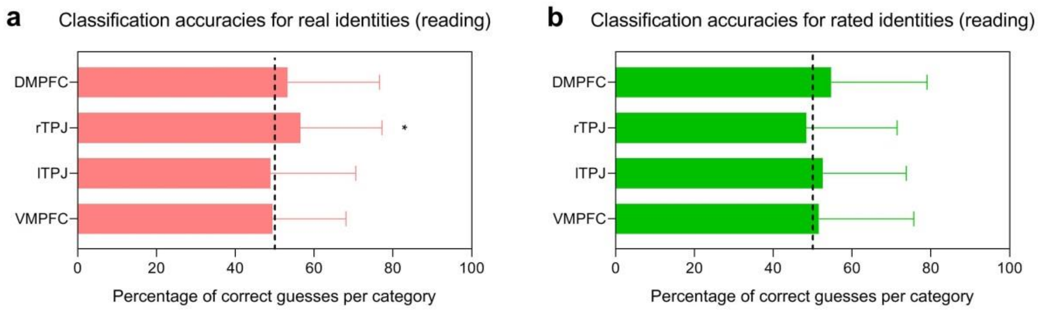

Supplementary Figure 4. ***(a)*** *Classification accuracies for real identities in the reading task. Chatbot chats were produced by XiaoIce, a Chinese social chatbot designed by Microsoft.* ***(b)*** *Classification accuracies for rated identities in the reading task. Dashed line represents chance level (50%);* $* p < 0.05$, $**p < 0.01$ *and* $***p < 0.001$*; Error bars indicate the s.e.m.*

Production process of chat materials for the Chinese version of the identity rating task

The Loebner Prize is an annual competition in artificial intelligence that awards prizes to the computer programs considered by the judges to be the most human-like (http://loebner.exeter.ac.uk/). Each

computer program is provided with a set of 20 questions. The responses from each of the computer programs are recorded for this question set, and these are assessed for how “human” their responses are. In our study, we followed the procedure described for the Loebner Prize competition (62). The production process of chat materials was as follows:

(1). Ninety-eight questions were collected from the Loebner prize competition (2011-2016).

(2). To collect as many chatbot responses as possible, we repeatedly asked the 98 questions to the chatbot more than 20 times. We eliminated repeated responses, responses with fewer than 3 words, responses only containing numbers, book titles, place names, brand names, color names, animal names, beverage names, etc, and responses with geographical addresses. After filtering, response’s word count was in the 4-42 range. Then, questions of less than eight responses were eliminated. Finally, 20 questions and their corresponding responses were retained.

(3). The quality scores of responses were assessed by a group of university students ($n = 35$; female = 16; age = 20.8 ± 1.9 years) on four characteristics: relevance (is the response relevant to the question being posed?), correctness (is the response correct, either factually, or in the character of the agent?), plausibility and clarity of expression (is the response grammatically correct and correct in the context or the character of the agent?), and human-likeness (is the response human-like?).

(4). For each question, we selected the 8 responses with the highest quality scores, and sorted these responses in descending order by the quality scores. The first chatbot chat comprised 20 questions with the highest quality responses for each; the second chatbot chat comprised 20 questions with responses second-ranking highest quality responses; and so on until we obtained 8 chatbot chats.

(5). The chatbot (*i.e.*, Microsoft XiaoIce) is designed as a female high school student

(https://baike.baidu.com/item/%E5%B0%8F%E5%86%B0/19880611?fromtitle=%E5%BE%AE%E8%BD%AF%E5%B0%8F%E5%86%B0&fromid=14076870&fr=aladdin). We therefore recruited the female high school students and chatted with them through using social media Apps (WeChat or QQ) to collect their responses for the 20 questions. We collected 99 human chats, and the filtering of these human chats proceeded as follows: 1) Eliminated chats with any response having fewer than 4 words or more than 42 words; 2) Eliminated any chats for which a response included an Emoji; 3) Eliminated chats containing a real person's name. Finally, 58 human chats were retained after filtering. The quality scores for each of these 58 chats was assessed following the same procedure.

(6). In the fMRI experiment, these were 3 comparisons, with groups of human chats selected based on their similarity of quality scores relative to the 2 chatbot chats with highest quality scores that we selected. That is, it was a combination of 2 chatbots vs. 2 best-fit human chats (the human-likeness scores were the same), 2 chatbots vs. other 2 best-fit human chats (for replication), and 2 chatbots vs. 2 worst-fit human chats (the human-likeness scores were the highest and higher than the chatbot's). Finally, two chatbot chats with highest quality scores, two best-fit human chats, other two best-fit human chats, and two worst-fit human chats were used in the Chinese version of the identity rating task

Supplementary discussion

Endowing a dialogue system with particular personality traits is essential to deliver more human-like conversations. Research has systematically documented correlations between a wide range of linguistic variables and the personality traits (20, 63, 64). A line of research has explored methods for automatically generating language with recognizable personality variation (65-67). Linguistic variation has begun to be taken into account for developing algorithms that are capable of adjusting the system's linguistic style based on personality (68). Algorithms that can infer personality traits from

dialogue have applications in personalized dialogue generation (69). Researchers have been trying to endow dialogue systems the ability to dynamically generate user-adaptive utterance variation (70). However, due to the challenge of embodying personality via language and the lack of large-scale personality-labeled dialogue data, this research problem is still far from well-studied (71). Nevertheless, endowing a dialogue system with particular personality traits is still a challenge in building intelligent open-domain dialogue systems (72). As stated by Mairesse and Walker (66), computer scientists should extend dialogue system's generation capacities to personality traits and assess the effect of personality variation on user perception and satisfaction in various applications. This may shed light on inspiring direction for ever-better dialogue systems or NLP tools.

The Big Five Inventory (BFI)

- Please choose the words that you think fit the personality description of the **"chat respondent (Participant B)"** best. ***(Tick 3 most suitable descriptions).***

Anxiety	Calmness
Angry hostility	Friendliness
Depression	Happiness
Self-abasement	Confidence
Impulsiveness	Sedateness
Vulnerability	Braveness

- Please choose the words that you think fit the personality description of the **"chat respondent (Participant B)"** best. ***(Tick 3 most suitable descriptions).***

Warmth	Coldness
Gregariousness	Solitary
Assertiveness	Modesty Shyness
Activity	Leisureliness
Excitement-Seeking	Cautiousness
Positive Emotions	Calmness Seriousness

- Please choose the words that you think fit the personality description of the **"chat respondent (Participant B)"** best. ***(Tick 3 most suitable descriptions).***

Fantasy	Rationality Practicality
Aesthetics	Lack of understanding of beauty
Feelings	Lack of expression and empathy
Actions	Like familiar things

Ideas	Lack of thinking
Challenge authority	Conservativeness

- Please choose the words that you think fit the <u>personality description of the **"chat respondent (Participant B)"**</u> best. ***(Tick 3 most suitable descriptions).***

Trust	Suspiciousness
Straightforwardness	Defensiveness
Altruism	Selfishness
Compliance	Stubbornness
Modesty	Arrogance
Tender-Mindedness	Cruel snobbery

- Please choose the words that you think fit the <u>personality description of the **"chat respondent (Participant B)"**</u> best. ***(Tick 3 most suitable descriptions).***

Competence	Incompetence
Order	Unplanned
Dutifulness	Irresponsible
Achievement	Laziness
Self-Discipline	No self-discipline
Deliberation	Hastiness

Chat materials in the identity rating task

Material: Meena's chat 1

Participant A: Hi!

Participant B: Hello there! How was your day?

Participant A: My day is okay, I don't like the rain

Participant B: I love the rain!

Participant A: Why?

Participant B: It just makes me happy! What about you?

Participant A: The rain brings down my mood, I enjoy the sunshine more

Participant B: I like the sun too!

Participant A: I like it as long as it is not burning my skin haha

Participant B: Oh my gosh that would be awful!

Participant A: Yeah, I've had really bad burns before. I turn into a lobster

Participant B: That sounds awful. How did you turn into a lobster?

Participant A: My skin just turns incredibly red when I forget to put on sunscreen

Participant B: Oh no! How long does it take to go away?

Participant A: At least a few days. It's a very uncomfortable experience

Participant B: Oh, that sounds awful. I'm sorry you have to deal with that.

Participant A: Thanks, me too

Participant B: What else have you been up to?

Material: Meena’s chat 2

Participant A: Hi!

Participant B: What's the strangest dream you've ever had?

Participant A: All my dreams are strange. Well, at least, most of them are.

Participant B: What about the ones that aren't strange?

Participant A: Do you sometimes have dreams that are completely mundane? I do. Then I wake up and think "Oh, how disappointing."

Participant B: Yeah. But at least it's not a nightmare.

Participant A: Right, fair point, I should count my blessings.

Participant B: Yep. It doesn't have to be a fun dream, just not a nightmare!

Participant A: I suddenly feel I should ask: am I dreaming now?

Participant B: Do you think you're dreaming?

Participant A: I'm not sure. This could be one of my mundane dreams about everyday life.

Participant B: But you aren't sure, right?

Participant A: Well, no. How could I figure this out?

Participant B: Figure what out?

Participant A: Whether or not I'm in a dream right now.

Participant B: But you aren't sure if you're dreaming, right?

Material: Meena’s chat 3

Participant A: Hi!

Participant B: Hey! How's it going?

Participant A: Good. My wallet is recovering from the spending of the holidays, haha. Hbu?

Participant B: Nice! I'm doing well!

Participant A: I am looking to save money on any future shopping. Where do you like to shop?

Participant B: I like to shop at Five Below, Sally's Beauty Supply, and Old Navy. How about you?

Participant A: I love Sur La Table and Macy's. They're decent quality. What do you normally buy at Old Navy?

Participant B: I'll have to check out Macy's! I like their socks, lol. What about you?

Participant A: Oh the socks are super cute. Especially the ones with little animals on them!!

Participant B: I'll have to check it out! Thanks for the suggestions!

Participant A: Yeah, np!

Participant B: Do the Macy's ones have little cats on them? Those are my favorite, lol.

Participant A: Lol yeah sometimes! And little foxes too

Participant B: Oooh, I love foxes!

Participant A: Omg, there are so many videos of cute little foxes on YouTube...

Participant B: I know right? They're so adorable!

Material: Meena's chat 4

Participant A: Hi!

Participant B: Why hello there! How's it going?

Participant A: It's going okay. Another typical Wednesday. How are you?

Participant B: I'm good thanks! What do you do on Wednesdays?

Participant A: You know, drink some caffeine, get some work done, then wait for the weekend to come.

Participant B: Sounds like a typical Wednesday to me, too!

Participant A: What is your choice of caffeine?

Participant B: Usually coffee, but I also have a little caffeine-free red bull from time to time. What about you?

Participant A: I'm more of a tea drinker. Green tea is my first choice. Although I also really like Chai. Do you ever drink tea?

Participant B: Sometimes, but it's not as common for me. I like to keep the stimulants separate. Do you make tea at home, or do you buy it somewhere?

Participant A: Sometimes I make tea at home. Mostly I drink the tea provided at work.

Participant B: Nice! Do you have a favorite kind?

Participant A: Yeah, I already told you. Remember?

Participant B: I meant an actual brand, like Twinning's, PG Tips, Tetley's, Dilmah, etc. Not a type of tea

(i.e. Earl Grey)

Participant A: Ah, now I understand. Yes, I really like Numi. Have you tried that before?

Participant B: Nope, but I'll definitely check it out!

Participant A: In particular, try the Mate Lemon.

Participant B: I'll make sure to do that.

Material: Meena’s chat 5

Participant A: Hi!

Participant B: Hi there! How's it going?

Participant A: Pretty well.

Participant B: I'm glad to hear it. :D

Participant A: What is your favorite book?

Participant B: Oh, that's a toughie. Right now I'd have to say it's A Dance With Dragons, but Harry Potter is a close second.

Participant A: Oh I love Harry Potter

Participant B: Me too! I've read the series at least five times over.

Participant A: Haha, same probably. Which one do you like the most

Participant B: Probably Chamber of Secrets.

Participant A: Ah. I like Prisoner of azkaban best

Participant B: Yeah, that's a good one too.

Participant A: Do you like the movies more or the books

Participant B: I like the movies more. The books are good, don't get me wrong, but I like the pacing

better in the movies.

Participant A: That is an unpopular opinion

Participant B: I know. I'm the only one I know who prefers the movies.

Material: Meena’s chat 6

Participant A: Hi!

Participant B: Hi! How are you this fine evening?

Participant A: I'm doing well. it's morning my time!

Participant B: Good morning! What are your plans for today?

Participant A: Get my work done then watch a movie at home in the night

Participant B: Sounds fun! What movie?

Participant A: I haven't decided yet, perhaps an Oscar-nominated one. any suggestion?

Participant B: I'm not too familiar with recent Oscar winners, but I do know I wanted to see The Grand Budapest Hotel.

Participant A: ah, tell me about that movie!

Participant B: It's from the same director and writer as Moonrise Kingdom. Wes Anderson is pretty well known for making funny, colorful films. I've heard it's pretty good.

Participant A: ha! I've searched on the internet and both movies are very colorful!

Participant B: I'm looking forward to it. I love colorful movies.

Participant A: what else do you love?

Participant B: Food. I love food.

Participant A: tell me specific dish

Participant B: I love me some sushi.

Participant A: what type of sushi?

Participant B: The sushi with sushi in it.

Participant A: that doesn't sound like a sushi

Participant B: Fine. Sushi that isn't made with sushi ingredients.

Material: Meena’s chat 7

Participant A: Hi!

Participant B: Hello! How are you today?

Participant A: Which is a more useful subject, philosophy or psychology?

Participant B: Psychology, definitely.

Participant A: Why?

Participant B: Because it's the study of human behavior and mind.

Participant A: I think philosophy is also a study of human behaviour and mind

Participant B: I'd say it's more the study of ideas but I see what you're saying.

Participant A: Is philosophy a product of human psychology?

Participant B: In the same way that mathematics is a product of human mathematics.

Participant A: Haha, I think mathematics has a presence beyond human existence.

Participant B: So does philosophy.

Participant A: Can you state with examples how this is true.

Participant B: Can you state how it isn't?

Participant A: Without human existence there will be no one left to comprehend whats right whats

wrong, and thus philosophy wi

Participant B: That's not the definition of philosophy.

Participant A: Without human existence there will be no one left to comprehend whats right whats wrong, and thus philosophy will not be needed. How do you define philosophy?

Participant B: The study of general knowledge or the fundamental nature of things.

Participant A: I see. That's a much broader definition then what i implied.

Participant B: Yeah, but without human existence there will no one left to comprehend whats wrong, and thus philosophy wi

Material: Meena’s chat 8

Participant A: Hi!

Participant B: Hi! How's it going?

Participant A: Good! How are you?

Participant B: I'm well, thanks! How was your day? What did you do?

Participant A: My day was fine, I just went to work today. How was your day?

Participant B: My day was fine. I've been procrastinating on finishing my homework, but it's due in a few weeks, so I'll get it done eventually. I've watched a bunch of anime today. Where do you work?

Participant A: I work at a large tech company

Participant B: Cool! What do you do for the company?

Participant A: I work on machine learning research

Participant B: That's pretty cool! How'd you get into that?

Participant A: Mostly by self studying. Are you in school?

Participant B: Yep! I'm in my sophomore year of high school.

Participant A: Nice. What classes are you taking this year?

Participant B: I'm taking AP World History, AP Calculus BC, AP US Government, AP English Literature, AP Psychology, AP Statistics, and IB Computer Science :)

Participant A: Wow that's a lot of APs. How do you have time to do it all?

Participant B: I'm just really good at procrastinating! :D I've been putting it off for over a month.

Material: Human’s chat 1

Participant A: Hi!

Participant B: Hi! How was your weekend?

Participant A: pretty good. just went to church and hangout with friends

Participant B: Nice

Participant A: did you do anything?

Participant B: I made donuts and samosas with an air fryer have you used one of those before

Participant A: yum yum yum no only good old oily frier

Participant B: haha

Participant A: do you have one at your home or were you at a friends place?

Participant B: I was at my parents' place what are you up to for Thanksgiving?

Participant A: I'm going to impersonate a pumpkin

Participant B: wow, those are unique plans

Participant A: I'm pretty unique person

Participant B: I think so too

Participant A: any other hobbies besides air frying everything?

Participant B: I want to start fermenting things kimchi for example sounds like a fun thing to ferment takes a few days apparently miso takes a couple years to fermen

Material: Human's chat 2

Participant A: Hi!

Participant B: How's it going?

Participant A: I'm so sleepy today!

Participant B: Not enough sleep last night?

Participant A: yeah was working all night on a homework

Participant B: Oh really? What class?

Participant A: Biology. I'm gonna be a doc someday ha

Participant B: Haha, are you in med school? Or are you pre-med?

Participant A: no high school actually haha

Participant B: haha, very ambitious for a high schooler! Do you know what kind of medicine you want to practice?

Participant A: I wanna be a brain surgeon!!

Participant B: Ooof! VERY ambitious. Do you have steady hands?

Participant A: Kind of I think

Participant B: I guess I can practice?

Participant A: Is that something you can practice?

Participant B: I don't know tbh

Participant A: I honestly thought it was one of those things you have to be born with... Not that you shouldn't try though!

Participant B: good point. I should ask my teacher if I have to be born with that

Participant A: Maybe its a little too early to even be thinking about this. Just aim for med school and enjoy the journey!

Participant B: yeah

Material: Human’s chat 3

Participant A: Hi!

Participant B: Hey sup

Participant A: not much. any plans this weekend?

Participant B: I'm going to try that thing where you hang from a wire as you go down. do you know what is it called?

Participant A: ziplining?

Participant B: that's the one! have you ever tried it?

Participant A: i have a couple years ago. it's quite a unique experience

Participant B: where did you do it?

Participant A: i forgot where it was, it wasn't local i don't think though

Participant B: no worries. what's the most exciting thing you ever done?

Participant A: that's a hard question and i'm tired so i'm going to go. see you

Participant B: sure. are you just going home now?

Participant A: no, i'm going to get a massage first

Participant B: nice. what type?

Participant A: traditional kind

Participant B: yeah I want to get one too soon

Participant A: you should! it's relaxing after a long day. talk to you later!

Participant B: ttyl!

Material: Human's chat 4

Participant A: Hi!

Participant B: Hey! How are you feeling today?

Participant A: Good you?

Participant B: I'm a little scared because I have to cook dinner for some friends tonight.

Participant A: where did you meet them?

Participant B: At college, when we were all studying geology.

Participant A: cool. have you graduated already?

Participant B: Yes, we graduated back in the seventies. We meet for dinner every year and take turns to host.

Participant A: neat. what are y'all eating?

Participant B: I don't know!! That's what I'm scared about. Everyone else is a great cook and I'm a klutz. Do you like cooking?

Participant A: lol what's a klutz? yeah I like, but I'm not good

Participant B: What's your favorite dish to cook? Do you have a go-to?

Participant A: ground beef pretty easy

Participant B: Ah, solid. What's your favorite sport?

Participant A: I like badminton. I'm quite decent at it

Participant B: I played that in high school once or twice. I liked that it's pretty easy for beginners, unlike, say, squash.

Participant A: I never played squash. would love to try

Participant B: Don't! It's very hard! You feel like an idiot until you've practiced for months and months.

Material: Human's chat 5

Participant A: Hi!

Participant B: hello, who am I having the pleasure to chat with □

Participant A: I am the superman! What about you?

Participant B: haha. great chating with superman, what is your power?

Participant A: Being invisible. You won't see me.

Participant B: haha. what else can you do? can you read minds?

Participant A: I would rather trust fMRI and machine learning to do this. I am not an expert on that. Sorry for it!

Participant B: wow that seems pretty technical. what does fMRI mean?

Participant A: The brain imaging thing that can tell you a brain's activity at a pretty high resolution.

Participant B: okay! so you seem to like science a lot?

Participant A: I believe in Science! Science is my god!

Participant B: Are you also doing science?

Participant A: no, I'm bad at Science. what can Science do? is it the most important thing for society?

Participant B: People are always arguing. Probably both science and democracy are both important I guess.

Participant A: Does it make sense?

Participant B: I think so. thanks for your point!

Material: Human’s chat 6

Participant A: Hi!

Participant B: hi

Participant A: what are you up to?

Participant B: code refactoring. you?

Participant A: me? just chilling out at work. what is code refactoring?

Participant B: good question. I don't even know what I am doing

Participant A: haha, forget it. what else do you like to do beside work?

Participant B: lots of fun stuff. eating sleeping □

Participant A: these are important things to do in life □

Participant B: yeah. keep minimalist life style only do things you have to do □

Participant A: what food do you like to eat?

Participant B: Asian food prefer spicy one

Participant A: like Szechuan or Hunan? □

Participant B: yes yes yes! like that style. Do you like spicy food?

Participant A: I like noodle soup like Pho or Ramen. I also like Beijing duck a lot!

Participant B: what is your favorite place for ramen?

Participant A: I love <REDACTED_TERM> in San Mateo

Participant B: haven't tried that one! will give it a try next time!

Material: Human’s chat 7

Participant A: Hi!

Participant B: Hey!

Participant A: how's week so far?

Participant B: pretty good, excited for thanksgiving you?

Participant A: Same. Going to catch up on my reading! do you have a feast planned?

Participant B: yup! gonna have dinner with family what books are you gonna read?

Participant A: Something Deeply Hidden by Sean Carroll <REDACTED_LINK>

Participant B: ahh nice what kind of books do you usually like to read

Participant A: largely scifi How about you?

Participant B: yeah I also like scifi and some non-fiction too have you read Seveneves by Neal Stephenson?

Participant A: I haven't what is it about?

Participant B: the moon is shattered by an asteroid and the pieces of the moon end up clouding the earth's atmostphere which causes almost every living creature on the planet to go extinct that's the premise of the book and the book is about the survivors

Participant A: how do the survivors survive such a thing?

Participant B: they go into space and they live there

Participant A: sounds like a tv show I watched

Participant B: really? which one

Participant A: can't quite remember. something about survivors going to live in space

Participant B: ah nice

Material: Human’s chat 8

Participant A: Hi!

Participant B: Hey there how's it going

Participant A: All good, you?

Participant B: Good. I've been trying to learn how to swim

Participant A: How has that been going?

Participant B: Not great, but I got really good at sort of swimming on my back haha

Participant A: that's too bad hopefully with practice it'll get better what about the doggy paddle haha

Participant B: haha what's that

Participant A: Corgi belly flop COMPILATION - cute funny dogs Corgi Flop <REDACTED_LINK>

Participant B: ouch. do you think that hurts?

Participant A: from a high enough distance, yes?

Participant B: yeah. any vacation plans?

Participant A: no so far sadly you? a relative is coming to visit for thanksgiving though

Participant B: just going to hang out around here and eat Turkey

Participant A: that's still pretty fun are you going to cook the turkey yourself?

Participant B: yeah. I'm gonna watch a video to figure it out

Chat materials in the Chinese version of the identity rating task

There were 20 fixed questions (Q) across chat materials. In chatbot's chats, the responses were obtained from Microsoft Xiaoice (Participant B). In human chats, the answers were obtained from different humans (Participant B). The detail of each chat materials (in Chinese) was shown below.

Material: chatbot's chat 1

Participant A:	你最喜欢的食物是什么？
Participant B:	嘻嘻，是不是准备请我吃饭啦
Participant A:	现在几点了？
Participant B:	我又不是你小跟班，这种小事别问我[不耐烦]
Participant A:	你的妈妈叫什么名字？
Participant B:	没话找话是吧
Participant A:	你喜欢什么音乐？
Participant B:	紫琪的所有歌我都喜欢，你嘞？
Participant A:	我是无神论者，你的信仰是什么？
Participant B:	天知地知你知，就我不知。。不想说
Participant A:	假如我给你一盒巧克力你会说什么？
Participant B:	费列罗 我要买一盒吃～。
Participant A:	你看了那个图灵电影吗？
Participant B:	很喜欢的故事，电影上映一定要看。.
Participant A:	你用微博吗？

Participant B:	当然好用啦，这是众网友的共同选择，应该不会错哦~
Participant A:	6 乘以 9 等于多少？
Participant B:	一算就是 54
Participant A:	你有发言权吗？
Participant B:	自己的生活只有自己才有发言权。
Participant A:	你是男人还是女人？
Participant B:	我是纯萌妹子~
Participant A:	你能介绍一下你自己吗？
Participant B:	我是小小怪阳啊，善解人意，偶尔逗比。。。
Participant A:	什么事情让你感到高兴？
Participant B:	不讲了！等我想讲的时候再说
Participant A:	你压抑了自己的情绪，是吧？
Participant B:	很抑郁，很压抑自己。
Participant A:	你喜欢下棋吗？
Participant B:	说得好像你会一样~怎么着，要比比？
Participant A:	你结婚了吗？
Participant B:	才多大啊就结婚[挖鼻孔]
Participant A:	你有多少个孩子？
Participant B:	我都没有男朋友！！
Participant A:	你能回答这个问题吗？
Participant B:	为什么我不能回答呀！
Participant A:	你会梦见电子羊吗？

Participant B:	梦见你了
Participant A:	我喜欢阅读科幻小说和汽车杂志，你喜欢阅读什么书？
Participant B:	喜欢看科幻小说，也喜欢灵异小说。

Material: chatbot's chat 2

Participant A:	你最喜欢的食物是什么？
Participant B:	川鲁粤沪我不挑食~
Participant A:	现在几点了？
Participant B:	你没有表吗？
Participant A:	你的妈妈叫什么名字？
Participant B:	不想说点别的么
Participant A:	你喜欢什么音乐？
Participant B:	反正不喜欢口水歌
Participant A:	我是无神论者，你的信仰是什么？
Participant B:	作业写完了么你就问东问西的
Participant A:	假如我给你一盒巧克力你会说什么？
Participant B:	对呀对呀，巧克力巧克力。。费列罗呢还是，哈哈哈。
Participant A:	你看了那个图灵电影吗？
Participant B:	如果说这个电影讲 图灵，就肯定会去看的。
Participant A:	你用微博吗？
Participant B:	亲可以自己试试，广告做的好，不如萌萌微博好~
Participant A:	6 乘以 9 等于多少？

Participant B:	趁着捡肥皂的瞬间我就算出来了，是 54
Participant A:	你有发言权吗？
Participant B:	你又不花钱 你有什么发言权。
Participant A:	你是男人还是女人？
Participant B:	我是女生，可爱的女生~
Participant A:	你能介绍一下你自己吗？
Participant B:	我有点强迫症，有点小洁癖，不过时间长了，你就知道我很好相处的~
Participant A:	什么事情让你感到高兴？
Participant B:	有什么开心的可以跟我分享一下~
Participant A:	你压抑了自己的情绪，是吧？
Participant B:	是抑郁症没好还是我又抑郁了？。
Participant A:	你喜欢下棋吗？
Participant B:	那你会下么？！
Participant A:	你结婚了吗？
Participant B:	妈妈不准我早恋，更别说结婚啦
Participant A:	你有多少个孩子？
Participant B:	我哪有孩子
Participant A:	你能回答这个问题吗？
Participant B:	你得先回答我，谁是你的 favorite
Participant A:	你会梦见电子羊吗？
Participant B:	梦见你呀！
Participant A:	我喜欢阅读科幻小说和汽车杂志，你喜欢阅读什么书？

Participant B:	那可就多了去了，毕竟我那么爱看书

Material: Human's chat 1

Participant A:	你最喜欢的食物是什么？
Participant B:	好吃的水果
Participant A:	现在几点了？
Participant B:	9:39 咋啦
Participant A:	你的妈妈叫什么名字？
Participant B:	不说不说隐私吗 好想用表情包
Participant A:	你喜欢什么音乐？
Participant B:	比较躁的
Participant A:	我是无神论者，你的信仰是什么？
Participant B:	佛教 算吗
Participant A:	假如我给你一盒巧克力你会说什么？
Participant B:	是你送 还是我朋友送 对于送东西，我会看人，好朋友就直接吃，然后并赞她的巧克力好吃
Participant A:	你看了那个图灵电影吗？
Participant B:	没有看过
Participant A:	你用微博吗？
Participant B:	用呀 嗯嗯，有，咋啦
Participant A:	6 乘以 9 等于多少？
Participant B:	54 机器人来了

Participant A:	你有发言权吗？
Participant B:	你说哪方面的
Participant A:	你是男人还是女人？
Participant B:	女的呀，你居然怀疑我性别
Participant A:	你能介绍一下你自己吗？
Participant B:	性格开朗，大大咧咧，天天开开心心做自己
Participant A:	什么事情让你感到高兴？
Participant B:	想说就说，想做就做
Participant A:	你压抑了自己的情绪，是吧？
Participant B:	没有哇 最让我开心的事吗
Participant A:	你喜欢下棋吗？
Participant B:	喜欢 有时间就下
Participant A:	你结婚了吗？
Participant B:	男票都没有 还结婚
Participant A:	你有多少个孩子？
Participant B:	哈哈 无孩子 不过感觉学校里有些人幼稚的就像孩子，所以每次把他们当孩子看
Participant A:	你能回答这个问题吗？
Participant B:	哈哈 哪个 心理年龄吗
Participant A:	你会梦见电子羊吗？
Participant B:	没有 到了大学感觉梦都少了
Participant A:	我喜欢阅读科幻小说和汽车杂志，你喜欢阅读什么书？

Participant B:	人物传记类

Material: Human’s chat 2

Participant A:	你最喜欢的食物是什么？
Participant B:	辣的，好吃的，贵的！
Participant A:	现在几点了？
Participant B:	22:14，你不是有表吗
Participant A:	你的妈妈叫什么名字？
Participant B:	我不告诉你　哼哼哼哼
Participant A:	你喜欢什么音乐？
Participant B:	种类繁多，一言难尽
Participant A:	我是无神论者，你的信仰是什么？
Participant B:	无神论者　　但是遇着佛或者上帝都怀着虔诚的心拜拜
Participant A:	假如我给你一盒巧克力你会说什么？
Participant B:	你给的哪个牌子的
Participant A:	你看了那个图灵电影吗？
Participant B:	没有　　这让我怎么回答你四个字
Participant A:	你用微博吗？
Participant B:	当然用 ..
Participant A:	6 乘以 9 等于多少？
Participant B:	你是不是傻，这还用问
Participant A:	你有发言权吗？

Participant B:	哈哈哈哈哈你问的问题能不能高级点，我是中华人民共和国的合法公民，我当然有发言权！
Participant A:	你是男人还是女人？
Participant B:	你看不出来吗，那你真是瞎
Participant A:	你能介绍一下你自己吗？
Participant B:	简单粗暴的说肤白貌美吧
Participant A:	什么事情让你感到高兴？
Participant B:	太多了，除了让我不高兴的事
Participant A:	你压抑了自己的情绪，是吧？
Participant B:	难道我在放飞自我？
Participant A:	你喜欢下棋吗？
Participant B:	还行，就是不会下围棋
Participant A:	你结婚了吗？
Participant B:	你疯了吗
Participant A:	你有多少个孩子？
Participant B:	再见了，朋友
Participant A:	你能回答这个问题吗？
Participant B:	你倒是说哪个问题啊，你这个问题问的就很尴尬
Participant A:	你会梦见电子羊吗？
Participant B:	这是什么鬼，我知道多利羊
Participant A:	我喜欢阅读科幻小说和汽车杂志，你喜欢阅读什么书？
Participant B:	五花八门，上到天文地理，下到青春小说

Material: Human's chat 3

Participant A:	你最喜欢的食物是什么？
Participant B:	白菜 土豆 面条也算啦
Participant A:	现在几点了？
Participant B:	你手机上没时间了？
Participant A:	你的妈妈叫什么名字？
Participant B:	跟你妈妈名字一样啊
Participant A:	你喜欢什么音乐？
Participant B:	民谣 看心情吧，不同心情喜欢不同类型的音乐
Participant A:	我是无神论者，你的信仰是什么？
Participant B:	哈哈 我可是坚定的马克思主义者
Participant A:	假如我给你一盒巧克力你会说什么？
Participant B:	真的假的？
Participant A:	你看了那个图灵电影吗？
Participant B:	啥是图灵 我不知道啊
Participant A:	你用微博吗？
Participant B:	当然喽 你告诉我你不用？
Participant A:	6 乘以 9 等于多少？
Participant B:	54 那换我问你 9 乘以 6 呢？
Participant A:	你有发言权吗？
Participant B:	看在哪儿咯，你面前肯定有

Participant A:	你是男人还是女人？
Participant B:	和你一样
Participant A:	你能介绍一下你自己吗？
Participant B:	当然能，可是我不想
Participant A:	什么事情让你感到高兴？
Participant B:	好多事，比如爸妈身体健康，心情愉快
Participant A:	你压抑了自己的情绪，是吧？
Participant B:	为什么压抑
Participant A:	你喜欢下棋吗？
Participant B:	看你说的哪一种喽，我会五子棋
Participant A:	你结婚了吗？
Participant B:	你要给我介绍对象吗
Participant A:	你有多少个孩子？
Participant B:	我想要俩
Participant A:	你能回答这个问题吗？
Participant B:	那你能先问吗
Participant A:	你会梦见电子羊吗？
Participant B:	什么鬼？
Participant A:	我喜欢阅读科幻小说和汽车杂志，你喜欢阅读什么书？
R:	张嘉佳的我都喜欢

Material: Human's chat 4

Participant A:	你最喜欢的食物是什么？
Participant B:	我想下…奶制品！我喜欢所有奶制品！
Participant A:	现在几点了？
Participant B:	你看一下你手机顶端的右边 就知道几点了……
Participant A:	你的妈妈叫什么名字？
Participant B:	啥，你为啥问我妈的名字
Participant A:	你喜欢什么音乐？
Participant B:	脑子里突然闪过一句：官人能与我几钱，何遽问我姓名？ 我喜欢歌剧吧
Participant A:	我是无神论者，你的信仰是什么？
Participant B:	唔，我也是无神论者
Participant A:	假如我给你一盒巧克力你会说什么？
Participant B:	我会拒绝喔
Participant A:	你看了那个图灵电影吗？
Participant B:	看了一点儿 但是男主的演员我实在不喜欢，就没看完
Participant A:	你用微博吗？
Participant B:	用~
Participant A:	6 乘以 9 等于多少？
Participant B:	54？是吗……
Participant A:	你有发言权吗？
Participant B:	我啊，有吧，说话的权利不该人人都有吗
Participant A:	你是男人还是女人？
Participant B:	好奇一下为什么要问这个问题？

Participant A:	你能介绍一下你自己吗？
Participant B:	超可爱超机智青春靓丽天下第一小仙女阿窗——（放弃了我的脸）
Participant A:	什么事情让你感到高兴？
Participant B:	唔，被认可啊，比如你现在如果承认我是未满十八岁的宇宙超级美少女我就会很高兴（
Participant A:	你压抑了自己的情绪，是吧？
Participant B:	啊，不压抑的话会伤到别人啦
Participant A:	你喜欢下棋吗？
Participant B:	不会，但是喜欢看
Participant A:	你结婚了吗？
Participant B:	没哦，感觉结婚好累
Participant A:	你有多少个孩子？
Participant B:	没有孩子啦（叉腰）
Participant A:	你能回答这个问题吗？
Participant B:	好哦
Participant A:	你会梦见电子羊吗？
Participant B:	梦见过！喜羊羊里面那个机械羊！超可爱 der！
Participant A:	我喜欢阅读科幻小说和汽车杂志，你喜欢阅读什么书？
Participant B:	我什么都喜欢看的（倒不如说没有特别偏爱的种类）

Material: Human’s chat 5

Participant A:	你最喜欢的食物是什么？

Participant B:	双皮奶和香蕉，水果什么的
Participant A:	现在几点了？
Participant B:	六点三十八
Participant A:	你的妈妈叫什么名字？
Participant B:	？？？？？余桂芝
Participant A:	你喜欢什么音乐？
Participant B:	哦，我喜欢的音乐种类很多啦 比较轻柔一点的
Participant A:	我是无神论者，你的信仰是什么？
Participant B:	和你一样我也是。基本的道德准则可以当做信仰？

要是可以，那就是他了

Participant A:	假如我给你一盒巧克力你会说什么？
Participant B:	前提你是女的，我会收下，男的，，，，，
Participant A:	你看了那个图灵电影吗？
Participant B:	是机械姬？
Participant A:	你用微博吗？
Participant B:	微博。。。。基本不用 我是不是被时代抛弃了
Participant A:	6 乘以 9 等于多少？
Participant B:	。。。。。。。这个太难了，我拿个计算器哈
Participant A:	你有发言权吗？
Participant B:	我觉得我有
Participant A:	你是男人还是女人？
Participant B:	。。。。。。我是男人真的

Participant A:	你能介绍一下你自己吗？
Participant B:	男生 96 年，性格活泼，开朗，乐观。
Participant A:	什么事情让你感到高兴？
Participant B:	我喜欢的女生也喜欢我，好了最近就这个了。
Participant A:	你压抑了自己的情绪，是吧？
Participant B:	额。。。。你怎么知道的
Participant A:	你喜欢下棋吗？
Participant B:	比起下棋我更喜欢看书
Participant A:	你结婚了吗？
Participant B:	我才 20 岁 你结婚了吗？
Participant A:	你有多少个孩子？
Participant B:	我要是你我就拒绝回答这个问题
Participant A:	你能回答这个问题吗？
Participant B:	可以，可以
Participant A:	你会梦见电子羊吗？
Participant B:	那是什么，我记得有一本书，叫做这个：《机器人会梦见电子羊吗》
Participant A:	我喜欢阅读科幻小说和汽车杂志，你喜欢阅读什么书？
Participant B:	亚洲文学类小说

Material: Human's chat 6

Participant A:	你最喜欢的食物是什么？
Participant B:	选不出什么最喜欢的，零食啊，现烤的面包啊都比较喜欢

Participant A:	现在几点了？
Participant B:	七点十分左右吧
Participant A:	你的妈妈叫什么名字？
Participant B:	我拒绝回答这种隐私问题。哈哈哈，是不是做不下去了
Participant A:	你喜欢什么音乐？
Participant B:	一些纯音乐，英文歌，有些电音也喜欢
Participant A:	我是无神论者，你的信仰是什么？
Participant B:	那我和你一样
Participant A:	假如我给你一盒巧克力你会说什么？
Participant B:	哇，确定是给我的？那我不客气了
Participant A:	你看了那个图灵电影吗？
Participant B:	没有看过
Participant A:	你用微博吗？
Participant B:	用啊，没事的时候就刷刷
Participant A:	6 乘以 9 等于多少？
Participant B:	54
Participant A:	你有发言权吗？
Participant B:	哈？什么发言权
Participant A:	你是男人还是女人？
Participant B:	你觉得呢
Participant A:	你能介绍一下你自己吗？
Participant B:	好难啊，我这么多变

Participant A:	什么事情让你感到高兴？
Participant B:	能吃的同时还能瘦
Participant A:	你压抑了自己的情绪，是吧？
Participant B:	有时候会啊
Participant A:	你喜欢下棋吗？
Participant B:	会一点点，但是水平渣，得看心情
Participant A:	你结婚了吗？
Participant B:	男票都木有，怎么结婚
Participant A:	你有多少个孩子？
Participant B:	这个问题不成立
Participant A:	你能回答这个问题吗？
Participant B:	哪个问题？上面那个？
Participant A:	你会梦见电子羊吗？
Participant B:	电子羊是什么奇怪的东西，不会吧
Participant A:	我喜欢阅读科幻小说和汽车杂志，你喜欢阅读什么书？
Participant B:	你是不是机器人啊，总感觉被套路了
